# Effectiveness of an Innovative Card Game as a Supplement for Teaching Factual Content to Medical Students: A Mixed Method Study

**DOI:** 10.7759/cureus.47768

**Published:** 2023-10-26

**Authors:** Gayatri Muthiyan, Payal Kasat, Vinu Vij, Ranjan S Solanki, Kirubhanand C, Bharat Sontakke

**Affiliations:** 1 Anatomy, All India Institute of Medical Sciences Nagpur, Nagpur, IND; 2 Anatomy, Dr B C Roy Multispecialty Medical Research Center, Kharagpur, IND; 3 Physiology, All India Institute of Medical Sciences Nagpur, Nagpur, IND; 4 Community Medicine, All India Institute of Medical Sciences Nagpur, Nagpur, IND

**Keywords:** small group discussion, pharyngeal arches, medical education, factual, card game

## Abstract

Introduction

Lectures and small group teaching are useful to transfer conceptual knowledge. Anatomy is the foundation of medical sciences, but it is perceived to be difficult to comprehend and recall. For such clinically relevant aspects of medical education that require memorization, educational card games can be very effective. As the complex concepts and terminology of Embryology are difficult to follow and retain, we designed a card game “MedFc” for a topic on pharyngeal arches. This study was planned to determine the effectiveness of the card game on curriculum comprehension, recall of factual topics among medical undergraduates, and its utility as a supplement to interactive lecture sessions.

Methods

The mixed method study was conducted in the Department of Anatomy of an undergraduate medical college. Ethical approval was obtained prior to beginning the study. Convenience sampling was done. From a batch of 50 first-year medical students, a total of 40 students consented to participate in the study, 24 (60%) were males and 16 (40%) were female participants. A lecture on the pharyngeal arches was conducted for the entire batch of 50 first-year medical students. After three weeks, the students who consented to participate in the study were randomly grouped into two groups of 20 each. The groups were the game group (which played the card game in teams of five) and the control group (which discussed the same topic in small groups of five). For both the group's pretests and posttests, 10 higher order multiple choice questions, were conducted and students’ feedback regarding the effectiveness of the teaching technique was obtained.

Results

Students opined that playing the card game was a superb experience, a positive use of time, and a very effective method of comprehension and memorizing complex topics. The scores increased from the pretest to the posttest indicating that both methods effectively reinforced the embryological concepts, but a t test showed that card game is more effective than small group discussions, with p-value = 0.008. The improvement in scores of students who had achieved <50% in pretest for the game group was statistically significant with t-value = 0.0023, when compared with the improvement in scores of similar students from the control group.

Conclusions

The study has demonstrated the effectiveness of “MedFc” card game in the recall of factual topics and can be used as supplementary material for enhancing learning amongst medical graduates. This educational card game applies gamification to Anatomy education to create a fun filled learning experience and is a valuable addition to the learning resources.

## Introduction

Lectures are useful for transferring knowledge and concepts to large groups. These are used to generate interest, explain essential concepts, provide basic knowledge, and direct further student learning. Small-group teaching aids effective learning among a limited number of participants. It helps the students to enhance their understanding of concepts, develop critical thinking, and acquire strategies for problem-solving [1].

Anatomy is considered to be the foundation of medical sciences. However, it is perceived to be a challenging subject in medical education, which is difficult to comprehend and recall. It has been observed that one common problem encountered by medical students is the difficulty in memorizing certain topics in order to understand the subject [2]. For such clinically relevant aspects in medical education that require memorization, educational card games can be designed, by integrating cognitive aspects of learning and game elements like achievements, intellectual challenges, scoring systems, etc., making the topic more interesting and meaningful, thereby enhancing and reinforcing students’ understanding of target content and concepts [3-6]. The use of well-designed, supplemental educational card games has potential benefits for the students involved in the learning process and also helps in developing problem-solving skills [7,8]. However, there are very few scientific studies on the effectiveness and usefulness of card games to enrich students’ learning in Anatomy.

The complex concepts and terminology of Embryology are considered onerous to conceive and retain. To improve students’ understanding and increase their interest in Embryology, an alternative method that is more interactive and student-centered is really needed. For instance, the structures developing from pharyngeal arches need to be memorized, prior to understanding the basis of related congenital anomalies. So, we designed a card game “MedFc“ for the topic on the development of pharyngeal arches, which could be suitably incorporated into the specific game design. This study was planned in order to determine 1) the effectiveness of the card game on curriculum comprehension, and recall of factual topics among medical undergraduates, and 2) its utility as a supplement to interactive lecture sessions.

## Materials and methods

The study was a mixed method approach with a pretest-posttest experimental design (with educational intervention) and students’ feedback to test the effectiveness of the card game, as the descriptive component. As the medical students form a vulnerable population, prior permission from the institutional ethics committee was obtained. A convenience sampling method was used. From a batch of 50 first-year medical students, a total of 40 students consented to participate in the study. A written informed consent was taken in a standard format from the students who agreed to participate in this study.

A lecture on the topic of pharyngeal arches was conducted for the whole batch of 50 first-year medical undergraduate students. Among the 40 students who participated in the study, 24 (60%) were males and 16 (40%) were female participants. After three weeks the participating students were randomly divided into two groups of 20 each. The groups were a game group and a control group. The facilitator introduced the topic and presented the summary of the topic in tabular format along with associated applied anatomy in both groups at the start. This information was to be recollected during the small group discussion and card game by the team members.

The students belonging to the game group played the “MedFc” card game for the development of pharyngeal arches, in teams of five members. Students in the control group discussed the same topic in small groups of five members. Pre and post-tests having ten higher order multiple choice questions were conducted and students’ feedback regarding the effectiveness of the teaching technique was obtained from both groups. After completion of the study, students from the control group were also given an opportunity to play the card game.

Game design

The development of pharyngeal arches was presented to the students in a tabular format along with the associated applied anatomy. The embryological terms for the derivatives of pharyngeal arches like muscles of mastication, facial muscles, stylopharyngeus, pharyngeal and laryngeal musculature, stapes, incus, malleus, lesser cornu of the hyoid, etc. were used and were appropriate to the level of students’ knowledge. Card size was appropriate (6cm x 8cm) and durable card sheets were used in the preparation of the cards. The deck of cards was compact and portable. A number of cards (25) was appropriate for five members in a group.

A pretest was conducted before starting the game in the initial five minutes. Each small group was given one set of the “MedFc” card games formulated for the development of pharyngeal arches. The directions for play were made clear and concise so as to be easily understood in the initial five minutes. These rules are exactly similar to the “Not at Home” card game rules played with the routine playing cards, as elaborated further. The role of the faculty was as a facilitator during the initial period for the introduction of the topic, summary table, and clarification of doubts, if required.

The specific requirement of the game was that the students should be able to recollect all the structures developing from the particular pharyngeal arch, for which he/she is holding cards and ask for the other cards of the same pharyngeal arch from the rest of the player at random as the leading player. If the player to whom a particular card is asked has the particular card, then he/she immediately surrenders the same to the lead player. And if he/she does not have the same, then he/she utters “Not at home” and continues the game further by asking for the components of the pharyngeal arch for which he/she holds the cards, by assuming the role of the lead player. Gradually the players were expected to collect all the pharyngeal arch cards from the various players by enumerating all the structures developing from the arches. The time allotted for the actual experience of the game by the teams was 45 minutes, followed by five minutes for the post-test. The length of time required to play the game was reasonable.

Data collection and analysis

Data obtained was analyzed using EpiData software (EpiData Association, Odense Denmark). Mixed-method analysis was implemented. The quantitative pre-posttest analysis to test the effectiveness of an educational card game in reinforcing embryological concepts in comparison with traditional small-group teaching methods was undertaken. Student t-test was used for analysis between groups. The p-value of <0.05 was considered statistically significant.

In the Indian context, a minimum of 50% score in professional examinations is essential for clearing these examinations. Hence, we have compared the pretest and posttest scores of students with reference to their pretest scores of <50% and >50% to determine the improvement in their performance. For analysis, the pretest scores of both groups were segregated into two segments taking 50% as a cut-off score. The posttest scores of these students were compared between the game and control groups.

A qualitative analysis of the students’ feedback was done. Four researchers independently examined the qualitative data for thematic analysis. Common themes were derived from the discussion and noted.

## Results

Comparison of the mean pretest scores for both groups is statistically insignificant, indicating the uniform distribution of students in the two groups based on their cognition of the topic (Table 1).

**Table 1:** Comparison of pretest and posttest scores and their difference between control group and game group.

	Pretest	Posttest	Difference in Posttest and Pretest scores
	Mean (SD)	Mean (SD)	Mean (SD)
Game group	4.48 (2.73)	8.68 (1.16)	4.2 (2.11)
Control group)	4.28 (1.79)	7.50 (1.48)	3.23 (1.32)
p value	0.7	0.008	0.09

The mean posttest scores were found to be increased for both game and control groups. This shows that both methods are effective in reinforcing embryological concepts. However, the game group showed statistically significant posttest scores when compared to the control group, p-value of 0.008. A greater difference was noted between pre and posttest scores in the game group than in the control group (Table 1).

The students who had <50% score in the pretest of the game group were found to have scored statistically significant posttest scores, with t-value = 0.0023, when compared with the post test scores of the students who had <50% score in the pretest of the control group (Table 2).

**Table 2:** Comparison of the difference in the percentages scores of posttest and pretest (for the students who had achieved scores < 50% and ≥ 50% in the pretest) between the control and game group. Two sample t-test with equal variance.

		Game group	Control group	Difference	t value
For scores < 50% in the pretest	Observations	9	12		
	Mean	58.89	37.5	21.39	0.0023
	Standard error	4.62	3.96		
	Standard deviation	13.87	13.73		
For scores ≥ 50% in the pretest	Observations	11	8		
	Mean	28.18	24.375	3.81	0.52
	Standard error	4.53	2.74		
	Standard deviation	15.05	7.76		

For the students who had achieved scores ≥ 50% in the pretest, there is no significant difference in the scores of the posttest and pretest between the groups (Table 2).

Improvement was observed in the posttest scores of all the students in the game group, while similar improvement was observed for a few students in the control group.

**Figure 1:**
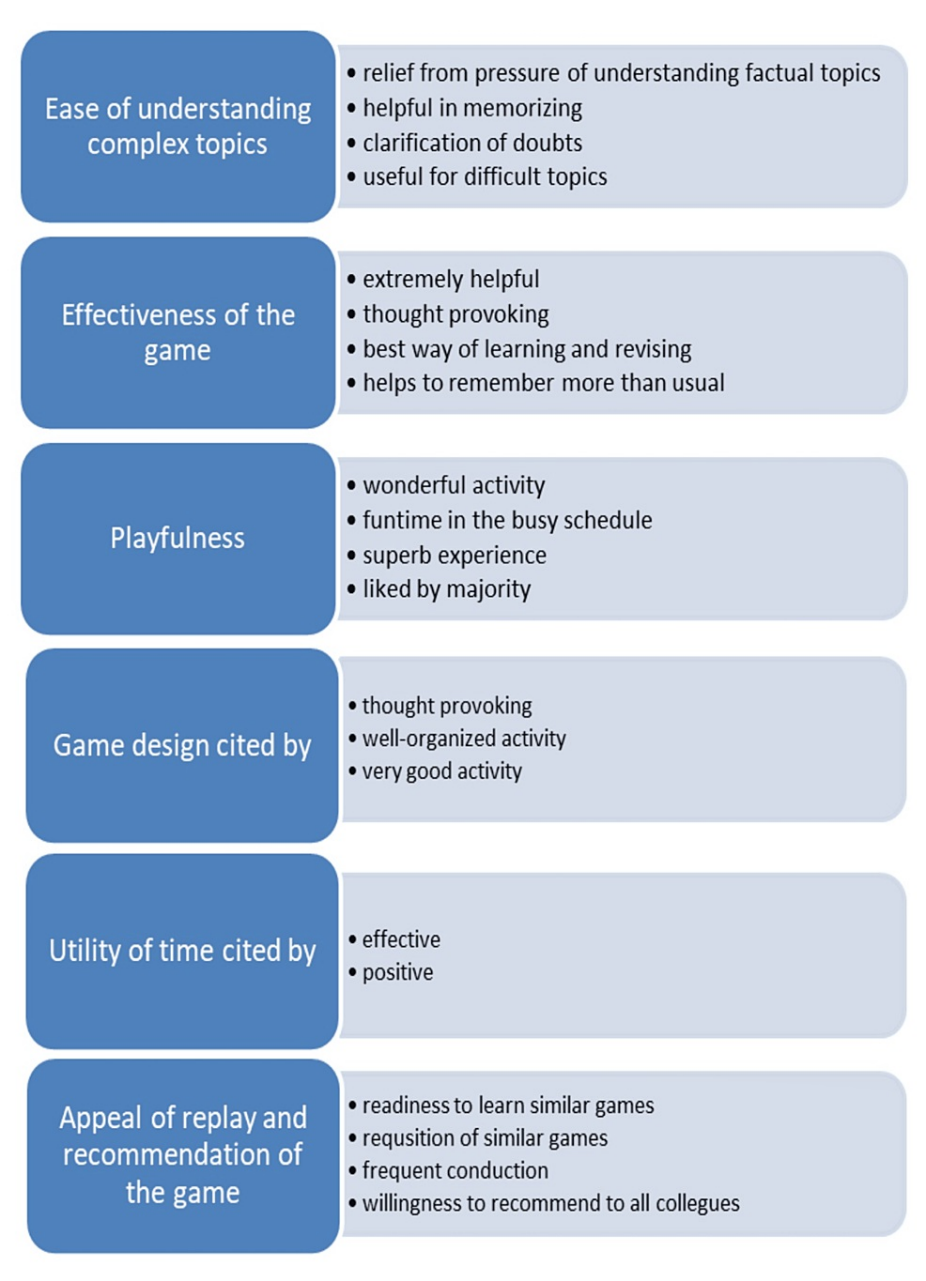
Salient themes from the feedback of the game group.

**Figure 2:**
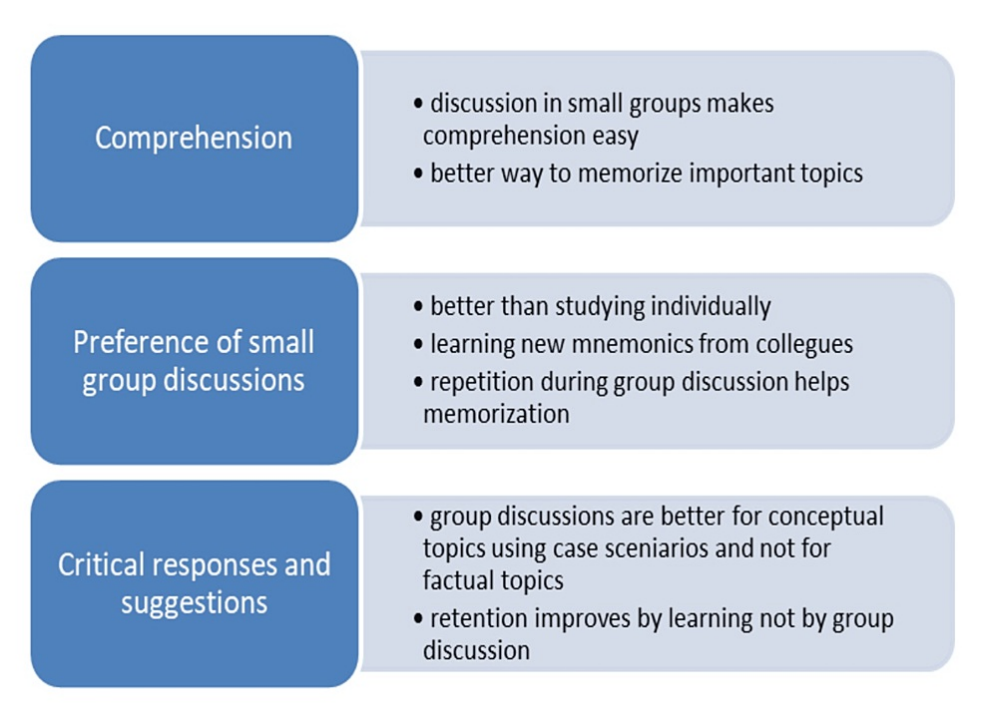
Salient themes from the feedback of the control group.

Some of the topics as suggested by the students that could be presented in the ‘MedFc’ card game format, are enumerated as follows: Extremities - muscles, joints, innervation, triangles, spaces, etc., neuroanatomy - cranial nerves, functional areas of the cerebral cortex, basal ganglia, thalamic nuclei, etc., thoracic region - development of the cardiovascular system, arteries, and veins, mediastinum, etc., head and neck region - ganglia, innervation of viscera, development of the eye, etc. Thematic analysis of the responses from the students’ feedback of the game group and control group are as stated in Figures 1, 2, respectively.

## Discussion

Comparison of the mean pretest scores for both groups was statistically insignificant (Table 1), indicating that the groups had similar prior knowledge of the topic.

Interest

In conformity with previous reports, the participants of the card game unanimously opined that the card game was effective in making factual topics enlivening (Figure 1), thus making grasp and recall easy [8-10].

Comprehension

The “MedFc” card game proved effective by way of easing the complicated process of learning factual embryology in an organized and engaging manner. This is clear from their verbatim responses “we need these activities on hard topics which help students be relieved from pressure of understanding such topics,” “this type of activity helps a lot,” etc. (Figure 1). These findings are consistent with the previous studies [8-10]. The feedback encourages the investigators to think about using similar interventions for more topics.

Playfulness

The playfulness of a game is influenced by the degree of uncertainty while playing the game, flexibility of decisions, the level of challenge, equal conditions for fair play, opportunities to compete with the team members or to cooperate based on the needs of the game, and the level of interactivity. The students' feedback conveyed the enjoyment and excitement they experienced and their appreciation for the design of the game. The students opined the game to be a very good, thought-provoking, and well-organized activity (Figure 1). The playability and playfulness as evidenced by this study were consistent with the results of earlier educational game-based research [11-14].

Productive use of time

This game was played in the post-lunch session when usually the students tend to become inattentive and doze. The students expressed that playing the game was a positive and productive use of time. This is very important since such games have the potential to deliver essential concepts in a very active way, completely utilizing such relatively ineffective time slots. This is a unique finding of this study.

Replay

There was continuous appeal for replaying the game, which is clear from students' verbatim responses like “these activities should be done more times,” “please come up with more such games,” “will love to have more such activities and would learn them all,” etc. (Figure 1). With the appeal and endeavor of replay acquisition of mastery in content is possible [6]. Thus, after a session of lectures dedicated to learning the fundamental concepts, playing the game would be a way to supplement the material already taught [15,16]. A game designed for knowledge transfer has to be appealing and fun filled. A game that does not cause excitement and is boring cannot transfer knowledge [16]. Thus, our findings are in conformity with the previous studies.

Interaction

Card games have the advantage of face-to-face students’ interaction and can be played anytime, anywhere with physical cards. Learning through playing the educational card game requires students to be attentive, efficient, and respond quickly. Such interpersonal skills are vitally important in cognitive processes and planning during professional life while working in teams [17]. In conformity with the previous studies, the card game encouraged harmonious and active student interaction, and involvement. The students also agreed that the game is worth recommending (Figure 1).

Planning strategies

In order to master the game and win, players have to reflect on their prior knowledge, solve problems during the game process, and correlate various concepts [18]. This is clear from the students' verbatim response “before conducting such activity please inform the topic one day prior, so that we can prepare and play it well.”

Evaluation

The evaluation of medical games is fundamental since it is not necessary that a good game in itself yields good learning results. Hence, we incorporated a mixed-method analysis. A pre-post gaming knowledge evaluation and the players’ opinions with regard to the learning process were taken into consideration (Tables 1, 2).

The results of this study indicate that the card game is an effective educational tool for all students, including low achievers (pretest scores <50%) (Table 2). The game had a positive impact on students' cognitive domain, as measured by the posttest (Table 1). This suggests that the inclusion of such games in medical education resources can be a valuable way to improve comprehension and recall of factual topics, especially for students who struggle in these areas.

Small group discussion was also considered helpful for memorizing concepts and mixed responses regarding preference to group study versus individual study were obtained (Figure 2).

There is a wide range of technology in the form of e-textbooks, online applications, videos, etc., available to enhance the learning of human anatomy [2]. Such media-based applications make the students glued to their screens and they get easily distracted. Hence, card games in a physical form are preferred over their digital form and other online applications, videos, etc. However, the digitization of card games may be useful in the case of distant learning modules. It can also help students to carry out their learning anytime and anywhere.

Card games can entertainingly provide students with education. It provides them relief from the routine and has the full potential to simultaneously have a deep impact on their memories, especially in the retention and recall of certain clinically relevant facts. Many such games can be easily designed for various other topics in Human Anatomy and for other subjects, which can help future medical graduates grasp the essential concepts in a fun-filled manner.

Limitation

The limitation of the study was the use of convenient sampling techniques. Future studies with larger groups and other factual topics may be planned to further investigate and establish the effectiveness of the “MedFc” card game. For those with meager interest in playing cards, the game seemed to be an average activity. Hence, the game design can be made a little more complex, and more clinical aspects can be added as its components, in order to suit the needs of highly intellectual students.

## Conclusions

It is evident that the “MedFc” card game is effective in curriculum comprehension and recall of factual topics. Playing the game has also been considered as a positive and productive use of time by the participants. Such games give an enriching experience to medical students, as well as simplify learning. When incorporated into the medical curriculum, it can improve the learning process of the students, produce better results, improve their academic skills, and form the basis of improvement of their clinical skills. We propose its use as a supplementary material for enhancing learning of factual topics for undergraduate medical students.
